# Araguspongine C Induces Autophagic Death in Breast Cancer Cells through Suppression of c-Met and HER2 Receptor Tyrosine Kinase Signaling

**DOI:** 10.3390/md13010288

**Published:** 2015-01-08

**Authors:** Mohamed R. Akl, Nehad M. Ayoub, Hassan Y. Ebrahim, Mohamed M. Mohyeldin, Khaled Y. Orabi, Ahmed I. Foudah, Khalid A. El Sayed

**Affiliations:** 1 Department of Basic Pharmaceutical Sciences, School of Pharmacy, University of Louisiana at Monroe, 1800 Bienville Drive, Monroe, LA 71201, USA; E-Mails: mohamedreda_pharmacy@yahoo.com (M.R.A.); hassanyahia_1982@yahoo.com (H.Y.E.); mohyelmm@warhawks.ulm.edu (M.M.M.); A_foudah@hotmail.com (A.I.F.); 2 Department of Clinical Pharmacy, Faculty of Pharmacy, Jordan University of Science and Technology, Irbid 22110, Jordan; E-Mail: nmayoub@just.edu.jo; 3 Department of Pharmaceutical Chemistry, Faculty of Pharmacy, Health Sciences Center, Kuwait University, Safat 13110, Kuwait; E-Mail: kyorabi@hsc.edu.kw

**Keywords:** araguspongine C, autophagy, breast cancer, c-Met, HER2

## Abstract

Receptor tyrosine kinases are key regulators of cellular growth and proliferation. Dysregulations of receptor tyrosine kinases in cancer cells may promote tumorigenesis by multiple mechanisms including enhanced cell survival and inhibition of cell death. Araguspongines represent a group of macrocyclic oxaquinolizidine alkaloids isolated from the marine sponge *Xestospongia* species. This study evaluated the anticancer activity of the known oxaquinolizidine alkaloids araguspongines A, C, K and L, and xestospongin B against breast cancer cells. Araguspongine C inhibited the proliferation of multiple breast cancer cell lines *in vitro* in a dose-dependent manner. Interestingly, araguspongine C-induced autophagic cell death in HER2-overexpressing BT-474 breast cancer cells was characterized by vacuole formation and upregulation of autophagy markers including LC3A/B, Atg3, Atg7, and Atg16L. Araguspongine C-induced autophagy was associated with suppression of c-Met and HER2 receptor tyrosine kinase activation. Further *in-silico* docking studies and cell-free Z-LYTE assays indicated the potential of direct interaction between araguspongine C and the receptor tyrosine kinases c-Met and HER2 at their kinase domains. Remarkably, araguspongine C treatment resulted in the suppression of PI3K/Akt/mTOR signaling cascade in breast cancer cells undergoing autophagy. Induction of autophagic death in BT-474 cells was also associated with decreased levels of inositol 1,4,5-trisphosphate receptor upon treatment with effective concentration of araguspongine C. In conclusion, results of this study are the first to reveal the potential of araguspongine C as an inhibitor to receptor tyrosine kinases resulting in the induction of autophagic cell death in breast cancer cells.

## 1. Introduction

Receptor tyrosine kinases (RTKs) are key regulators of critical cellular processes including cell growth, differentiation, survival, and repair [1]. Multiple RTKs were identified for their oncogenic potential in breast cancer. It is well-established that aberrations in epidermal growth factor (EGF) receptor and HER2 signaling are associated with worse prognosis and more aggressive phenotypes of breast cancer [2]. Recently, strong evidence supports the role for the hepatocyte growth factor (HGF) and its receptor, c-Met, in the development and progression of breast carcinoma [2]. Abnormally increased expression of c-Met has been detected in human breast cancer and is associated with poor prognosis [3]. RTKs are often selectively altered on malignant cells. Therefore, they represent attractive targets for cancer therapy, with a number of agents already approved for clinical use [4].

**Figure 1:**
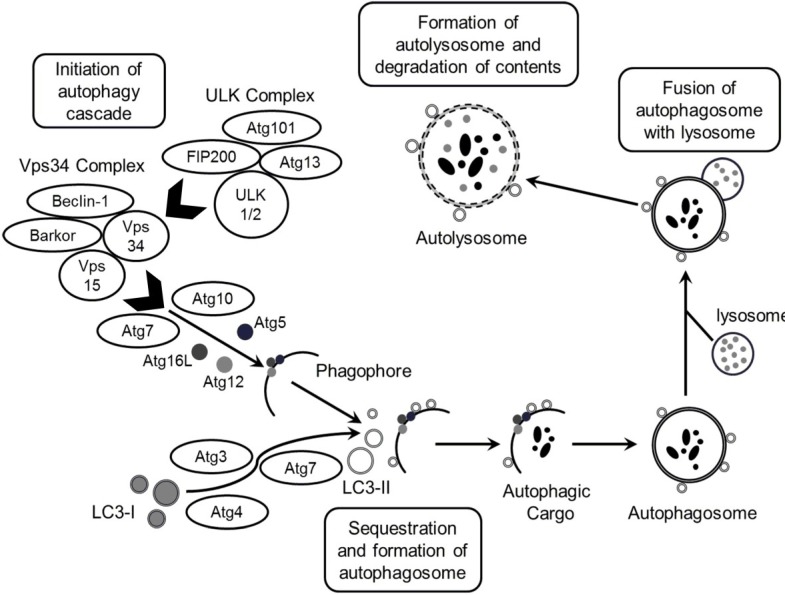
Schematic illustration of autophagy. The process of autophagosome formation consists of several stages, namely initiation, elongation and maturation and fusion. ULK complex (comprising ULK1/2−Atg13−FIP200−Atg101) is responsible for initiation of autophagy, in response to certain signals. In addition to initiation signals by the ULK complex, formation of double-layered membrane (phagophore) within the cytosol requires the action of the Vps34 complex (Vps34−Vps15, Beclin-1 (Atg6)−Barkor). The elongation stage requires cleavage of the microtubule-associated protein 1 light chain 3 (Atg8/LC3) by Atg4, resulting in the formation of cytosolic LC3-I protein, which is conjugated to phosphatidylethanolamine (PE) to form membrane bound LC3-II. In the meantime, the formation of Atg5–Atg12–Atg16L1 protein complex associates with the membrane and facilitates LC3 conjugation to PE and determines the sites of LC3 lipidation. The membrane grows to enwrap a portion of the cytosol, forming an autophagosome containing autophagic cargo. Next, lysosomes fuse with the autophagosome, releasing lysosomal hydrolases resulting in degradation of the vesicle contents and formation of autolysosome.

Autophagy is a catabolic process that digests cellular contents within lysosomes [5,6]. Autophagy is characterized by the formation of double-membrane vesicles, known as autophagosomes, which are engulfed by cytoplasmic molecules (Figure 1). Subsequently, the autophagosome fuses with the lysosome, which provides hydrolases and the sequestered contents undergo degradation and/or recycling (Figure 1). To date, the mammalian target of rapamycin (mTOR) is the most well-characterized autophagy regulator [7,8]. Recently, there has been growing evidence to suggest that decreased autophagic activity is related to tumorigenesis [6]. Therefore, induction of autophagic cell death may represent a promising tool for cancer cell eradication.

**Figure 2:**
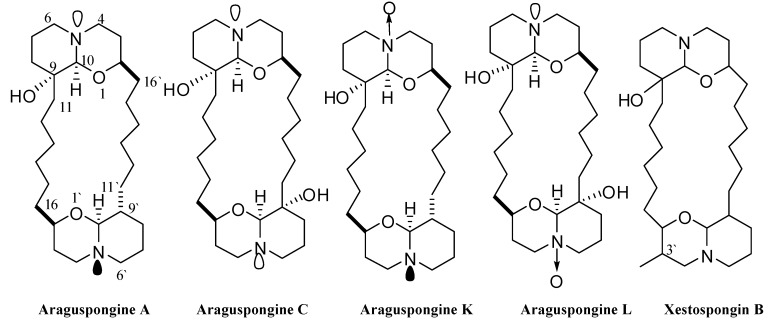
Chemical structures of araguspongines A, C, L, K, and xestospongin B.

Marine sponges are rich sources of bioactive, unique, and chemically diverse natural products [9]. Xestospongins are marine natural products first isolated from the Pacific sponge *Xestospongia exigua* (Kirkpatrick) [10]. Chemically, araguspongines/xestospongins are dimeric 2,9-disubstituted 1-oxaquinolizidines (Figure 2). Stereochemically, the *trans*-2,9-disubstituted 1-oxaquinolizidine rings predominantly maintain a *trans*-decaline-like conformation, unlike the *cis*-disubstituted rings, which adapt a *cis*-decaline-like conformer [10]. Conformational variations of araguspongine/xestospongin alkaloids can significantly affect their biological activities and molecular targets [11]. Bioactivities reported for araguspongines and xestospongins include vasodilatory [12], cytotoxic [13], antifungal [11], antimalarial and antituberculosis [10], as well as antiplatelet activities [14]. Xestospongins and araguspongines have been extensively evaluated for their potential to modulate calcium release by multiple cellular calcium channels [15,16,17]. Xestospongins/araguspongines are powerful inhibitors of inositol 1,4,5-trisphosphate (IP3) receptor, a calcium channel mainly located at the endoplasmic reticulum [15,16,17].

Some marine-derived xestospongins and araguspongines were patented for modest antitumor activities in 1997 but little is known about their anticancer properties [18]. Therefore, the goal of this study was to evaluate the anticancer activity of araguspongines in multiple breast cancer cell lines *in vitro* and to characterize the mechanisms associated with the anticancer activity of araguspongine C in breast cancer cells.

## 2. Results

### 2.1 Chemical Diversity of Tested Oxaquinolizidine Alkaloids and Their Effect on Breast Cancer Cell Viability

Five known oxaquinolizidine alkaloids (Figure 2) have been identified and screened for their anticancer activity using the HER2-overexpressing breast cancer cell line BT-474 cells. The structures represent diverse dimeric *trans*-2,9-disubstituted 1-oxaquinolizidines, with mono and dihydroxy substitutions (araguspongines A and C, respectively), *N*-oxides (araguspongines K and L), and monohydroxy and C-3′ methyl substitution (xestospongin B). Two day treatment of BT-474 cells resulted in antiproliferative activity and inhibition of breast cancer cell growth. The effect of oxaquinolizidine alkaloids treatment on breast cancer cells is shown in Table 1. Suppression of BT-474 cell viability was most remarkable with araguspongine A and araguspongine C treatments, with IC_50_ values of 9.3 and 15.2 μM, respectively (Table 1). Alternatively, xestospongin B and araguspongine L were the least active inhibitors of BT-474 cell growth when compared with other araguspongines used for this screening (Table 1).

**Table 1:** IC_50_ values for oxaquinolizidine alkaloids after 48 h treatment of BT-474 breast cancer cells in culture. Cells were plated at a density of 1 × 10^4^ cells per well in 96-well culture plates and maintained in RPMI-1640 media supplemented with 10% FBS and allowed to adhere overnight. The next day, cells were divided into different treatment groups and then given various treatments in RPMI-1640 medium containing 40 ng/mL HGF for 48 h. Viable cell number was determined using the MTT assay. IC_50_ values were calculated using non-linear regression curve fit analysis using GraphPad Prism software.

Compound	IC_50_ (μM) ± SEM
Araguspongine A	9.3 ± 3.2
Araguspongine C	15.2 ± 2.1
Araguspongine K	29.5 ± 3.8
Araguspongine L	35.6 ± 3.7
Xestospongin B	52.5 ± 4.5

### 2.2. Effects of Araguspongine C on Viability, Morphology, and Colony Formation of Breast Cancer Cells

**Figure 3:**
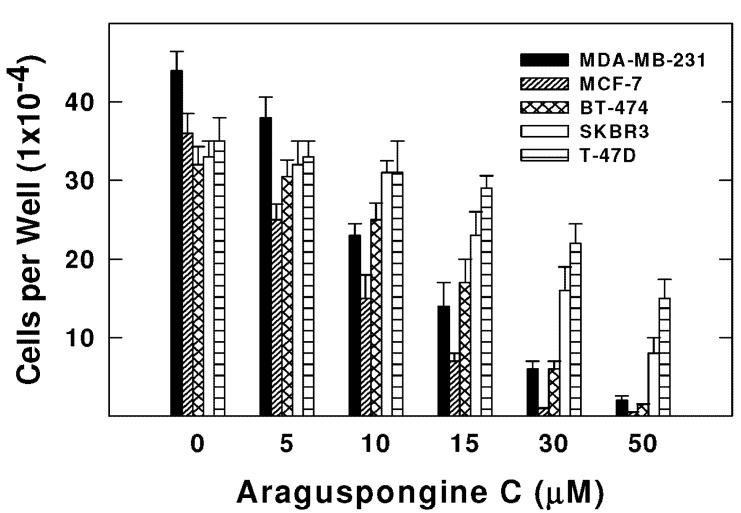
Effect of araguspongine C treatment on viability of breast cancer cell lines *in vitro*. MDA-MB-231, MCF-7, BT-474, SKBR3, and T-47D human breast cancer cells were plated at a density ~1 × 10^4^ cells per well in 96-well culture plates and maintained in RPMI-1640 media supplemented with 10% FBS and allowed to adhere overnight. The next day, cells were divided into different treatment groups and then given various treatments in RPMI-1640 medium containing 40 ng/mL HGF for 48 h. Viable cell number was determined using the MTT assay. The data represent the mean ± SEM for three independent experiments.

**Figure 4:**
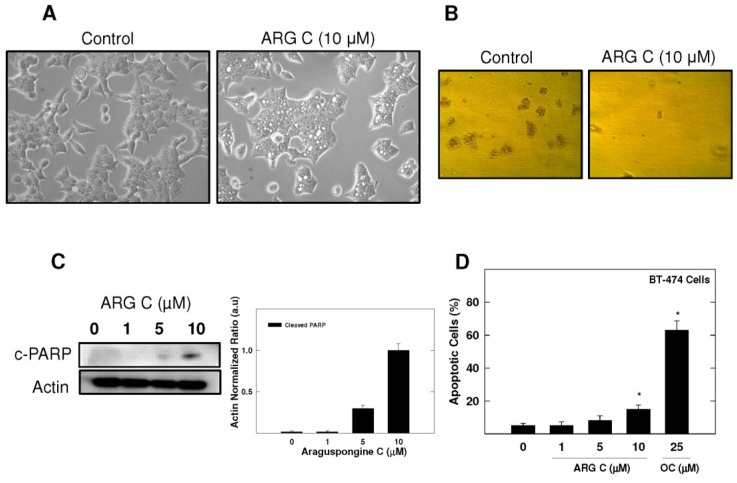
BT-474 breast cancer cells show characteristic vacuoles upon treatment with araguspongine C associated with cytotoxic effects. (**A**) Phase-contrast microscopy of BT-474 cells after araguspongine C treatment. BT-474 cells were treated with vehicle or araguspongine C at 10 μM for 24 h. The morphology of the cells was observed under an inverted phase contrast microscope. Magnification 100×; (**B**) Soft agar assay shows inhibition of BT-474 cell anchorage-independent growth by ARG C. BT-474 cells were cultured for 8 days in the absence (**left**) or presence (**right**) of 10 μM araguspongine C according to assay protocol and colony formation was observed under light microscope; (**C**) Western blot analysis of relative levels of c-PARP after araguspongine C treatment for 48 h in BT-474 breast cancer cells. Cells were plated at a density of 1 × 10^6^ cells/100 mm culture plate and maintained in RPMI-1640 media supplemented with 10% FBS and allowed to adhere overnight. The next day, cells were divided into different treatment groups and then given various treatments in RPMI-1640 medium containing 40 ng/mL HGF for 48 h. At the end of treatment period, cells were lysed and equal amounts of whole cell extracts were fractionated on SDS-PAGE gels and immunoblotted as described in Materials and Methods. The visualization of β-actin was used as a loading control. Representative blots are from one of the three experiments; (**D**) Flow cytometry analysis. Cells were plated at a density of 1 × 10^6^ cells/100 mm culture plates, allowed to attach overnight. Afterwards, cells were incubated in the respective control or araguspongine C-treated RPMI-1640 medium containing 40 ng/mL HGF for 48 h. Analysis of annexin V was determined using Annexin V-FITC Early Apoptosis Detection Kit as described in the Methods section. (−)-Oleocanthal was used as a positive control known to induce apoptosis at the dose used in this experiment. * *p* < 0.05 indicates values significantly different from non-treated cells. ARG C: Araguspongine C, c-PARP: cleaved Poly (ADP-ribose) polymerase, OC: (−)-Oleocanthal.

Araguspongine C exerted antiproliferative effects when applied to multiple breast cancer cell lines *in vitro*. Five breast cancer cell lines with different phenotypes and molecular characteristics were used for the evaluation of araguspongine C treatment over two days in culture. Results showed that it effectively suppressed the growth of all breast cancer cell lines used in a dose-dependent manner (Figure 3). However, growth inhibition of breast cancer cell lines showed a range of IC_50_ values (Table 2). MDA-MB-231 and MCF-7 cells were the most sensitive while T-47D cells were the least sensitive to the antiproliferative activity of araguspongine C (Table 2). Araguspongine C was not toxic in the non-tumorigenic MCF10A mammary epithelial cells at the used treatment doses. Interestingly, araguspongine C-induced antiproliferative activity in BT-474 cells was associated with a characteristic change of cellular phenotype indicated by vacuole accumulation in all colonies of BT-474 cells exposed to the compound treatment as compared to their vehicle-treated control group (Figure 4A). Vacuole accumulation in BT-474 cells was observed with araguspongine C concentration of 10 µM within three hours of treatment exposure. Furthermore, these alterations in cancer cell phenotype were exclusively noticed upon araguspongine C treatment of BT-474 cells, but not with other breast cancer cell lines concomitantly treated with the compound or with other oxaquinolizidine alkaloids previously used in BT-474 cell treatment.

**Table 2:** IC_50_ values for araguspongine C after 48 h treatment of multiple breast cancer cell lines in culture. IC_50_ values were calculated using non-linear regression curve fit analysis using GraphPad Prism software (GraphPad Software Inc., La Jolla, CA USA).

Cell Line	IC_50 _(µM) ± SEM
MDA-MB-231	10.1 ± 2.3
MCF-7	8.5 ± 1.6
BT-474	15.2 ± 2.1
SKBR3	18.3 ± 2.5
T-47D	46.1 ± 4.8

For further evaluation of araguspongine C effects on BT-474 cells, cytotoxic and anchorage-independent growth studies were considered (Figure 4B,D). Araguspongine C treatment at 10 µM concentration was able to inhibit BT-474 cell anchorage-independent growth in soft agar assay compared to the vehicle-treated control cells (Figure 4B). In addition, araguspongine C treatment at 10 μM concentration induced apoptosis (cell death) in BT-474 cells treated for 48 h as compared to their vehicle-treated counterparts. Apoptosis was assessed by measuring the levels of Poly (ADP-ribose) polymerase (PARP) cleavage as shown by Western blot results (Figure 4C). Moreover, araguspongine C-induced cell death was additionally confirmed by determination of annexin V (apoptotic marker) and PI (oncotic marker) binding using flow cytometry in BT-474 cancer cells (Figure 4D). Araguspongine C at a concentration of 10 µM resulted in modest increase (17%) for the number of apoptotic cells (annexin V-positive) when compared to 25 μM (−)-oleocanthal (>60%) which was used as a positive control known to exert potent cytotoxic activity at the concentration used for this assay [19].

### 2.3. Autophagic Activity of Araguspongine C in BT-474 Breast Cancer Cells

**Figure 5:**
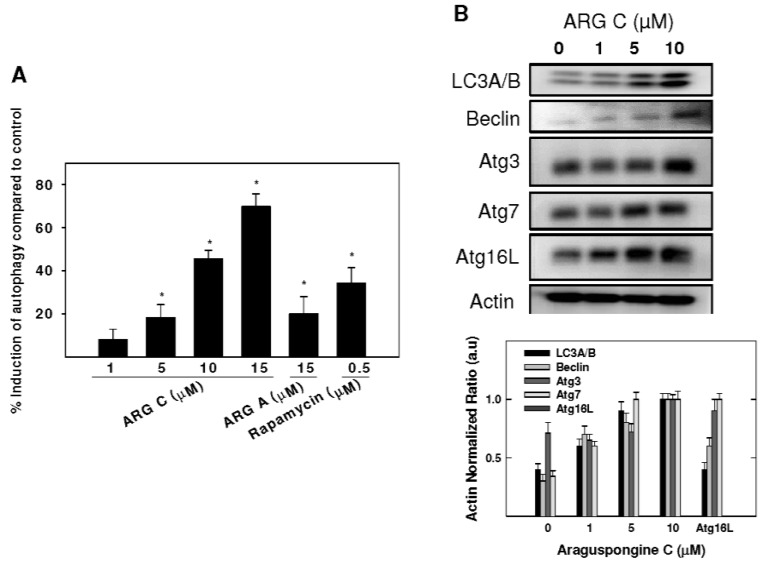
Araguspongine C-induced autophagy is associated with upregulation of autophagy-related proteins in BT-474 cancer cells. (**A**) Cyto-ID-coated autophagosomes in araguspongine C treated breast cancer cells. BT-474 cells were incubated with araguspongine C, araguspongine A or rapamycin (positive control) for 18 h and stained with Cyto-ID for 30 min at 37 °C. Intracellular Cyto-ID fluorescence was analyzed by microplate reader; (**B**) Western blot analysis of relative levels LC3A/B, Beclin-1, Atg3, Atg7, Atg16L after araguspongine C treatment for 24 h in BT-474 breast cancer cells. Cells were plated at a density of 1 × 10^6^ cells/100 mm culture plate and maintained in RPMI-1640 media supplemented with 10% FBS and allowed to adhere overnight. The next day, cells were divided into different treatment groups and then given various treatments in RPMI-1640 medium containing 40 ng/mL HGF for 24 h. At the end of treatment period, cells were lysed and equal amounts of whole cell extracts were fractionated on SDS-PAGE gels and immunoblotted as described in Materials and Methods. The visualization of β-actin was used as a loading control. Representative blots are from one of the three experiments. ARG C: Araguspongine C, ARG A: Araguspongine A.

A concentration of 10 μM araguspongine C caused accumulation of vacuoles in BT-474 cells and showed an increase of apoptotic cells. Therefore, the potential to induce toxic autophagy in BT-474 cells was examined. Cyto-ID Green reagent staining showed the relative fluorescence intensity of cells was increased in a dose-dependent manner, indicating the occurrence of autophagy (Figure 5A). Treatment with 5, 10, and 15 μM resulted in 18.2%, 45.5%, and 69.8% autophagy induction in BT-474 cells (Figure 5A). However, applied at the same concentration, araguspongine A showed a weaker autophagic activity in BT-474 cells (19.9%). In order to further evaluate the occurrence of cellular autophagy, Western blot studies were considered to assess araguspongine C effects on autophagy molecular modulators in BT-474 cancer cells. Treatment caused a dose-dependent increase in the total protein levels of LC3A/B, Beclin-1 (Atg6), Atg5, Atg7, and Atg16L1 in BT-474 breast cancer cells (Figure 5B). The increase in the expression of previously mentioned autophagy markers followed a dose-dependent manner and was clearly prominent at 10 μM. Taken together, these findings support the fact that araguspongine C molecular actions in BT-474 cells are mediated through the induction of autophagic cell death.

### 2.4. Effect of Araguspongine C on c-Met and HER2 Receptor Tyrosine Kinase Activation

**Figure 6:**
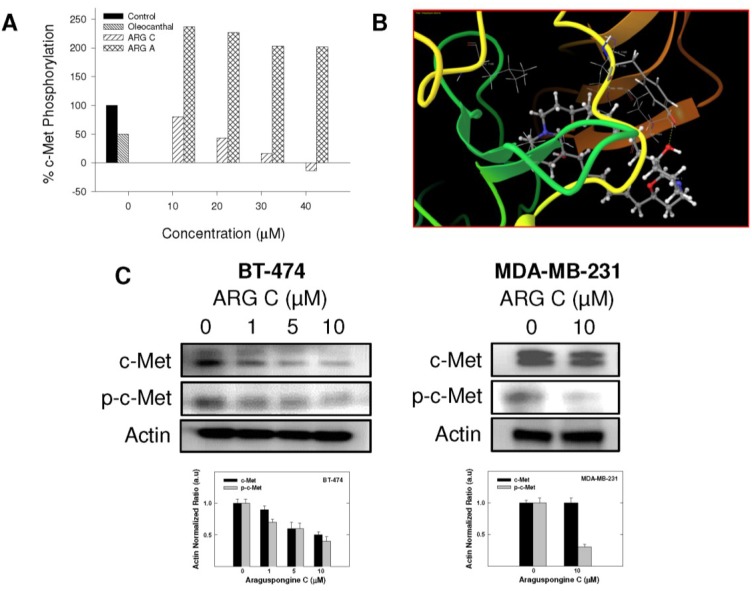
*In vitro* and *in-silico* c-Met receptor tyrosine kinase inhibition by araguspongine C. (**A**) Z-LYTE c-Met Kinase Assay. Araguspongine C was able to inhibit c-Met phosphorylation in a dose-dependent manner. 20 μL/well reactions were set up in 96-well plates containing kinase buffer, ATP, Z-LYTE Tyr6 Peptide substrate, c-Met kinase and, compound of interest as an inhibitor. After incubation at room temperature, development solution containing site-specific protease was added to each well. After another incubation period, the reaction was stopped, and the fluorescent signal was determined on a plate reader. (−)-Oleocanthal was used as a positive control in this experiment [21]; (**B**) *In-silico* binding mode of araguspongine C (ball and stick) in c-Met kinase domain (secondary structure and surface representation). Araguspongine C assumes a shallow U-shaped binding mode with partial wrapping around Met 1211 and the C-9′-hydroxyl on the oxaquinozolidine ring of araguspongine C contributes a critical single-point hydrogen bonding interaction with the side chain phenolic hydroxyl of Tyr 1159 in the hinge region. Araguspongine C’s six-carbon aliphatic linker tethered the dimeric oxaquinolizidine ring system participates in hydrophobic interactions with the side chains of Ile 1084, Val 1092, Ala 1108 and Leu 1140 in the hydrophobic sub-pocket; (**C**) Western blot analysis of relative levels of total c-Met and phosphorylated-c-Met (p-c-Met) protein levels after araguspongine C treatment for 48 h in BT-474 and MDA-MB-231 breast cancer cells. Treatment was done according to the previously described protocol [19]. The visualization of β-actin was used as a loading control. Representative blots are from one of the three experiments. ARG C: Araguspongine C, ARG A: Araguspongine A, p-c-Met: Phosphorylated c-Met.

Given the critical role of RTKs in controlling cell survival and death in response to external stimuli, we investigated the potential that araguspongine C-induced autophagic death to be associated with suppression of c-Met and/or HER2 signaling in BT-474 cancer cells. The Z-LYTE Kinase Assay-Tyr6 Peptide kit (Invitrogen) was used to assess the ability of araguspongines A and C to inhibit c-Met phosphorylation (activation) [19]. In these experiments, the olive phenolic (−)-oleocanthal was used as a positive standard control for activity comparison [20]. Araguspongine C was able to inhibit c-Met phosphorylation in a dose-dependent manner, with an IC_50_ value of 19.9 μM (Figure 6A). On the other hand, araguspongine A did not show significant inhibition of c-Met phosphorylation even when applied in concentrations up to 40 μM. The calculated IC_50_ of (−)-oleocanthal in this assay was 5.0 µM, which was consistent with the reported value (~4.8 µM) validating the results of this study [20]. The remarkable difference in the activity between araguspongines A and C as c-Met inhibitors can be explained based on their chemical structures and molecular modeling studies. Molecular modeling experiments were used to investigate the possible binding modes of araguspongines within the catalytic domain of unphosphorylated c-Met using the Schrödinger molecular modeling software package (Figure 6B). Araguspongine C assumed a shallow U-shaped binding mode with partial wrapping around Met 1211 at the c-Met kinase domain’s activation loop (Figure 6B). The C-9′-hydroxyl group of araguspongine C’s oxaquinozolidine ring participated in a critical single-point hydrogen bonding interaction with the side chain phenolic hydroxyl group of the Tyr 1159 at the hinge region (Figure 6B). This might explain, at least in part, its moderate micromolar activity level in Z-LYTE kinase biochemical assay. Additionally, the six-carbon aliphatic linker tethered the dimeric oxaquinolizidine ring system exerted hydrophobic interactions with the side chains of Ile 1084, Val 1092, Ala 1108 and Leu 1140 at the c-Met kinase domain hydrophobic sub-pocket. Alternatively, araguspongine A, which lacks C-9′-hydroxyl group, failed to satisfy such critical hydrogen bonding interactions within the hinge region and subsequently showed poor activity in the c-Met biochemical assay. These results indicated the importance of the C-9′-hydroxyl functionality in araguspongine C chemical structure for binding the kinase domain of c-Met promoting enhanced c-Met inhibitory activity compared to oxaquinolizidine alkaloids lacking this functionality. Western blot analysis for the total and phosphorylated levels of c-Met in BT-474 and MDA-MB-231 cancer cells was considered in order to validate the c-Met inhibitory activity of araguspongine C in breast cancer cell lines. Western blot experiments showed that araguspongine C treatment resulted in a dose-dependent reduction of the total c-Met levels with a subsequent decrease in phosphorylated (active) levels in BT-474 cells. Interestingly, araguspongine C treatment caused suppression of c-Met receptor activation (phosphorylation) without changing the total levels of the receptor in MDA-MB-231 human breast cancer cells (Figure 6C).

**Figure 7:**
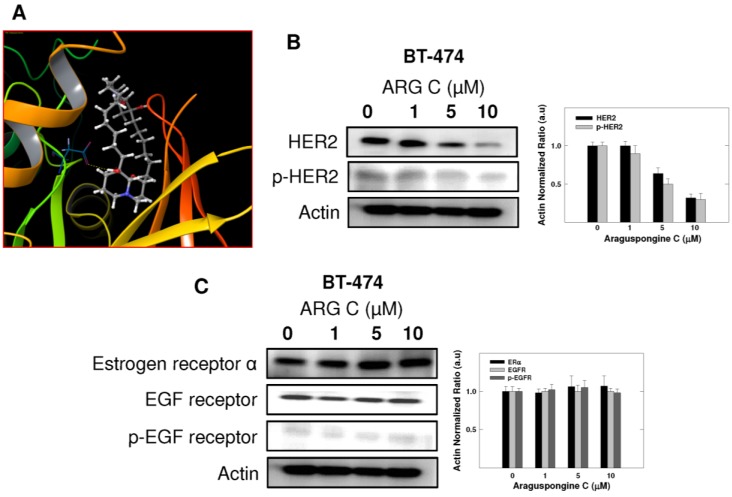
*In vitro* and *in-silico* ability of araguspongine C to downregulate HER2 levels and suppresses receptor activation in BT-474 breast cancer cell. (**A**) Molecular docking study of araguspongine C at the HER2 crystal structure. Docking studies suggested a hydrogen bonding interaction between araguspongine’s C-9′-tertiary hydroxyl group on the quinazolidine scaffold with the carboxylate side chain of Asp863 at the DFG motif; (**B**) Western blot analysis of relative levels of total and phosphorylated HER2 after treatment with araguspongine C for 48 h in BT-474 cancer cells. Treatment was done according to the previously discussed protocol [21]. The visualization of actin was used as a loading control. Representative blots are from one of the three experiments; (**C**) Western blot analysis of the relative levels of estrogen receptor, EGF receptor, and phosphorylated-EGF receptor after araguspongine C treatment for 48 h. Treatment was done according to the previously described protocol. The visualization of β-actin was used as a loading control. Representative blots are from one of three experiments. ARG C: Araguspongine C, p-HER2: Phosphorylated HER2, EGF receptor: Epidermal growth factor receptor, p-EGF receptor: Phosphorylated epidermal growth factor receptor.

BT-474 is a HER2-overexpressing breast cancer cell line. Thus, further docking studies were conducted for araguspongine C on the crystal structure of HER2. Molecular docking study of araguspongine C on HER2 crystal structure (PDB: 3RCD [22]) suggested a hydrogen bonding interaction between C-9'-tertiary hydroxyl group of the quinazolidine scaffold with the carboxylate side chain of Asp 863 in the DFG motif (Figure 7A). The DFG motif (Asp863-Phe864-Gly365) of HER2 is located at the regulatory activation loop of the ATP binding pocket and is critical for HER2 protein kinase activity [23]. In active kinase conformation, the DFG motif is oriented towards the bound ATP, with the carboxylate side chain of Asp 863 residue able to coordinate with the magnesium ions bound to the β- and γ-phosphate groups of the ATP [23]. While in the inactive conformation, the DFG motif is flipped in such a way that Asp 863 no longer coordinates magnesium ion in the catalytic cleft [24]. Additionally, the importance of hydrogen bonding interaction of araguspongine C with Asp 863 at the DFG motif was obvious when the C-9'-hydroxyl group was replaced by hydrogen as in araguspongine A. Therefore, C-9'-hydroxyl of araguspongine C is an important pharmacophoric group to retain HER2 inhibitory and anticancer activities. Western blot experiments showed that araguspongine C treatment resulted in a dose-dependent reduction of the total HER2 levels with a subsequent decrease in phosphorylated (active) levels in BT-474 cells, confirming the molecular modeling results (Figure 7B). Further expression studies in BT-474 cells revealed no alterations to the total and the phosphorylated (active) levels of EGF receptor in response to araguspongine C treatment (Figure 7C). Similarly, Western blot experiments to examine the effects of araguspongine C treatment (10 μM) in MDA-MB-231 cancer cells did not result in changes in the total and the phosphorylated levels of EGF receptor (data not shown). Lack of activity of araguspongine C towards EGF receptor in both BT-474 and MDA-MB-231 cell lines may suggest some degree of selectivity toward c-Met and HER2 kinases. In addition, Western blot results showed no alterations to the total levels of estrogen receptor in BT-474 cells treated with araguspongine C for two days in culture (Figure 7C).

### 2.5. Effect of Araguspongine C on PI3K/Akt/mTOR Signaling Pathway and IP3 Receptor Levels

**Figure 8:**
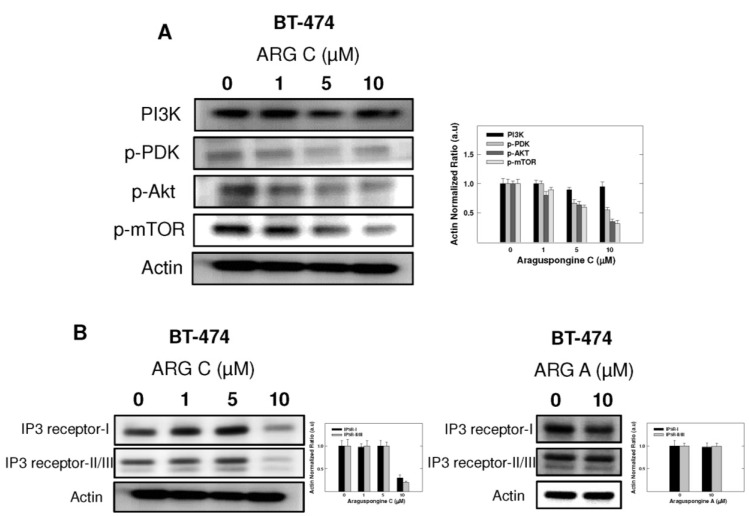
Cytotoxic autophagy induced by araguspongine C treatment in BT-474 cancer cells is associated with suppression of mTOR activation and downregulation of IP3 receptor levels. (**A**) Western blot analysis of relative levels of PI3K, p-PDK, p-AKT, and p-mTOR levels after araguspongine C treatment for 48 h. Treatment was done according to the protocol described previously. The visualization of β-actin was used as a loading control. Representative blots are from one of the three experiments; (**B**) Western blot analysis for the total levels of IP3 receptor-I/II/III upon treatment of BT-474 cells with araguspongine C or araguspongine A for 48 h in cell culture. Treatments were done according to the protocol described previously in the Methods section. The visualization of β-actin was used as a loading control. Representative blots are from one of the three experiments. ARG C: Araguspongine C, ARG A: Araguspongine A.

PI3K/Akt/mTOR signaling cascade is an essential pathway that is activated in response to ligand binding and activation of RTKs. Western blot studies showed that araguspongine C treatment caused a dose-dependent reduction of the levels p-PDK, p-Akt and p-mTOR in BT-474 cells (Figure 8A). In addition, araguspongine C treatment (10 μM) caused a reduction of total levels of IP3 receptor isoforms in BT-474 cells (Figure 8B). However, treatment of BT-474 cells with growth inhibitory concentration of araguspongine A (10 μM) did not cause a reduction of IP3 receptor level as compared to araguspongine C treatment (Figure 8B). These results suggest explanatory mechanisms to the autophagic activity of araguspongine C to be mediated, at least in part, through suppression of PI3K/Akt/mTOR pathway, and reduced IP3 receptor levels, which are both pathways recognized as major regulators of autophagy.

## 3. Discussion

Five known oxaquinolizidine alkaloids were tested for antiproliferative activity against the HER2-overexpressing breast cancer cell line BT-474 cells. Results suggest the need for two basic nitrogens of both araguspongine oxaquinolizidine rings for activity. This was based on the lower activity of the *N*-oxide-containing araguspongines K and L due to the loss of one oxaquinolizidine nitrogen lone pair of electrons to form the *N*-oxide bond. Xestospongin B also showed modest activity, possibly due to different oxaquinolizidine ring geometry and stereochemistry. Chemically, araguspongine C has C-9,9′-dihydroxylated groups, unlike the C-9 monohydroxylated araguspongines A and B. The additional C-9'-hydroxyl was virtually proved to be the main binding and anchoring pharmacophoric group at the c-Met and HER2 kinase domains (Figure 6B and Figure 7A). This may justify the additional c-Met and HER2 inhibitory activity as previously described in the Results section. Although araguspongine C’s basic nitrogens N-5 and N-5′ did not play direct binding roles, they ultimately play crucial roles in properly aligning binding pharmacophores at the c-Met and HER2 kinase domains. They certainly play critical binding roles at IP3 receptor since some xestospongins and araguspongines lacking hydroxyl groups were reported to be active IP3 receptor antagonists. Araguspongines and xestospongins with C-7 or C-9 oxaquinolizidine ring hydroxylated groups like araguspongine C, 9-hydroxyxestospongin C, and 7-hydroxyxestospongin A showed enhanced IP3 receptor antagonistic activity, inhibiting IP3-mediated calcium release, weakly inhibiting ryanodine receptor-1 but lacking activity toward endoplasmic/sarcoplasmic reticulum calcium-ATPase (SERCA) [16]. Results of this study demonstrated that the marine-derived araguspongine C treatment inhibited HGF-induced growth and proliferation of multiple breast cancer cell lines *in vitro*. Interestingly, growth inhibition observed in BT-474 cells was associated with the suppression of HER2 and c-Met RTK activity and subsequent induction of autophagy. Autophagic death was also associated with the suppression of PI3K/Akt/mTOR signaling pathway as well as a reduction in the total levels of IP3 receptor in BT-474 cancer cells.

HER2 is a proto-oncogene encoding for HER2 receptor tyrosine kinase. HER2 is amplified in 20%–25% of breast cancers leading to HER2 protein overexpression and aggressive tumor phenotype associated with reduced survival and high risk of metastasis [25]. c-Met is a high-affinity receptor for HGF expressed mostly in epithelial cells [21]. Solid clinical evidence showed that c-Met is overexpressed in 20%–30% of breast cancer cases and is a strong and independent predictor of decreased survival and poor patient outcome [26,27]. This study indicates that araguspongine C treatment resulted in dose-dependent antiproliferative effects in different breast cancer cell lines. Few studies had evaluated the anticancer effects of araguspongines in literature. Araguspongine E (xestospongin C) inhibited the growth of MCF-7 breast cancer cells stimulated by 5% fetal calf serum or 10 nM estradiol [28]. In addition, xestospongin D has been reported to inhibit the growth of human leukemia and breast cancer cell lines comprising the NCI panel [13]. Initial screening for the anticancer effects of five different known *bis*-1-oxaquinolizidine alkaloids in BT-474 breast cancer cells showed a spectrum of variable activity, with araguspongines A and C being the most effective inhibitors of cancer cell growth. Of these, araguspongine C was the only autophagy inducer of BT-474 cells and therefore was selected for further examination in a panel of breast cancer cell lines *in vitro*.

Autophagy can function as a unique caspase-independent mechanism of cell death that is distinct from apoptosis and necrosis [29]. Autophagy is characterized by the formation of double-membrane vesicles (autophagosomes) that engulf cellular components targeted for destruction (Figure 1) [29]. The important component proteins involved in the execution of autophagy have been grouped as autophagy-related proteins (Atgs) [8,30,31]. Nearly 30 autophagy-related genes are identified so far and are implicated in different stages of autophagy [6]. One of the complexes first involved in the autophagy process is the ULK complex which triggers autophagy upon the input of certain induction signals [32]. Next in the process, autophagosome formation requires the activity of class III PI3K as vacuolar sorting protein forms a complex with Beclin-1 (Atg6) [32]. The elongation stage requires cleavage of the microtubule-associated protein 1 light chain 3 (Atg8/LC3) by Atg4, resulting in the formation of cytosolic LC3-I protein, which is conjugated to phosphatidylethanolamine to form membrane bound LC3-II [31,32]. Eventually, autophagosomes fuse with lysosomes to form autolysosomes, in which their autophagic cargo is degraded by lysosomal proteases (Figure 1) [29,31]. Compared to apoptosis, features of autophagic cell death include massive autophagic vacuolization inside the cytoplasm and the absence of chromosome condensation and nuclear fragmentation [33]. Besides autophagic vacuoles, the specific features of autophagic cell death also include Beclin-1 (Atg6), Atg5, Atg12, or Atg7 involvement and LC3-I to LC3-II conversion [33]. This study clearly showed that araguspongine C treatment of BT-474 cells induced autophagic death as indicated by the accumulation of autophagic vacuoles inside the cells and upregulation of autophagic markers LC3, Beclin-1, Atg3, Atg7, and Atg16L (Figure 4 and Figure 5).

Autophagy is tightly regulated by upstream modulators, most essentially the PI3K/Akt/mTOR signaling pathway [30]. PI3K/Akt/mTOR signaling cascade is a major intracellular mediator of growth and survival [8,29]. Regularly, PI3K/Akt/mTOR signaling is activated by growth factors and nutrients; however, this pathway is constitutively active in many cancer types [29,30]. Since activation of the PI3K/Akt cascade promotes mTOR activity, this pathway is an important regulator of cellular autophagy. mTOR is known to suppress autophagy while promoting tumor cell growth, proliferation and survival [34]. Growth factor receptors such as c-Met and HER2 can activate PI3K/Akt/mTOR signaling cascade promoting cellular growth and survival. Earlier studies showed that inhibition of mTOR is sufficient to block cell survival induced by HGF/c-Met *in vitro* [35]. In the current study, araguspongine C treatment resulted in significant suppression of c-Met and HER2 receptor activation in BT-474 cancer cells. Further analysis showed that araguspongine C can directly inhibit c-Met and HER2 receptor activation (kinase domain phosphorylation) and that it can fit into the kinase domains of both c-Met and HER2 RTKs. In addition, suppression of c-Met and HER2 signaling by araguspongine C treatment resulted in the inhibition of PI3K/Akt/mTOR cascade, a major negative regulator of autophagy.

In cancer cell biology, autophagy is known to display a dual contrasting function [29,34,36]. Autophagic response can function as a protective mechanism allowing the recycling of proteins and cellular components to survive cell injuries induced by cytotoxic agents. Alternatively, cancer cells may undergo autophagic cell death following extreme autophagic degradation [36]. Recently, Han and colleagues identified potential interaction between Beclin-1 and HER2 in HER2-overexpressing BT-474 and SKBR3 breast cancer cells. Interestingly, the study identified a novel complex between HER2 and Beclin-1 that is disrupted by concurrent treatment with lapatinib resulting in cytoprotective autophagic response in these cells [37]. In contrast, other study showed that Ras-induced expression of Beclin-1 to promote autophagic cell death in MCF-7 breast cancer cells [38]. Thus, the induction of autophagic cell death may serve as a novel therapeutic strategy for eliminating cancer cells, especially those with high thresholds to apoptosis [34]. However, the complex role of autophagy in tumorigenesis requires specific pharmacological modulation of autophagy taking into consideration context- and cell-specific approaches [34]. In this study, exposure to araguspongine C resulted in a dose-dependent inhibition of breast cancer cell growth in culture, however, autophagic activity was a finding specific to BT-474 cells. These observations further confirm that induction of autophagy can vary based on cancer cell type and other potential settings.

**Figure 9:**
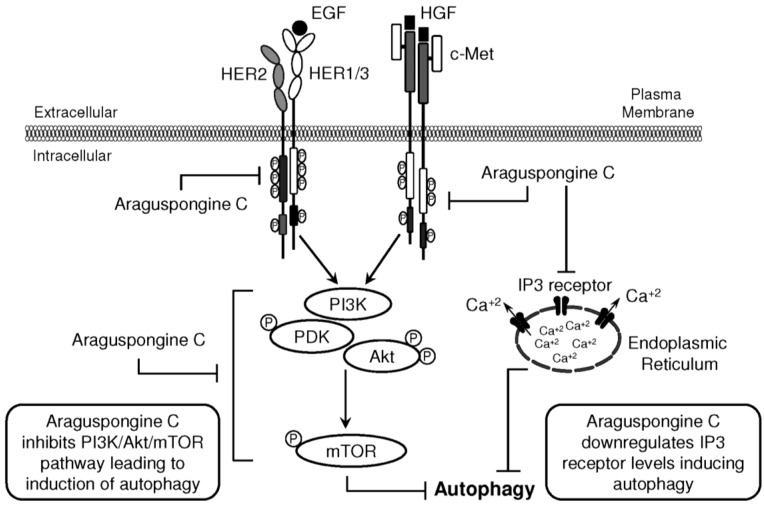
Schematic presentation for araguspongine C-induced autophagic death in breast cancer cells. Proposed direct inhibition of c-Met and HER2 receptor tyrosine kinases by araguspongine C. The inhibition of c-Met and HER2 activation in BT-474 breast cancer cells resulted in suppression of downstream molecular transducers, as indicated by suppression of PI3K/Akt/mTOR pathway. In addition, araguspongine C treatment resulted in a reduction in the total levels of IP3 receptor expressed by BT-474 cells. Taken together, araguspongine C inhibition of mTOR signaling pathway and inhibition of IP3 receptor resulted in the induction of autophagy in BT-474 cancer cells and subsequent inhibition of cell growth and proliferation.

Recent findings identified intracellular calcium as a key regulator of both basal and induced autophagy [31]. The inositol 1,4,5-trisphosphate (IP3) receptor, a calcium channel mainly located at the endoplasmic reticulum, plays a vital role in regulating calcium-dependent autophagy [7]. Identification of IP3 as a regulator of autophagy goes back to studies by Sarkar and colleagues who described an mTOR-independent pathway for regulation of autophagy in mammalian cells [39]. Consequent studies revealed that xestospongins and araguspongines are potent inhibitors of IP3 receptor [16,40]. In Hela cells, xestospongin B (araguspongine B)-induced autophagy was mediated through inhibition of IP3 receptor [15]. As shown in the Results section, araguspongine C treatment of BT-474 cells resulted in a reduction in the total levels of IP3 receptor expression at doses known to induce autophagic response (10 μM). Accordingly, it can be concluded that autophagic activity of araguspongine C in BT-474 cell line is mediated, in part, by downregulation of IP3 receptor levels as well as the inhibition of mTOR signaling pathway as (Figure 9) discussed earlier.

## 4. Experimental Section

### 4.1. Chemicals, Reagents and Antibodies

All chemicals and reagents were purchased from Sigma-Aldrich (St. Louis, MO, USA), unless otherwise stated. Araguspongines A, C, K, L and xestospongin B were isolated from the Red Sea sponge *Xestospongia exigua* (Kirkpatrick) (Demospongiae: Haplosclerida) and identified by spectral analyses [10]. A purity of >95% was established for each compound using ^1^H NMR and TLC analyses. Primary antibodies were purchased from Cell Signaling Technology (Beverly, MA, USA), except for IP3 receptor primary antibody which was purchased from Santa Cruz Biotechnology, Inc. (Dallas, TX, USA). Secondary antibodies were purchased from Cell Signaling Technology (Beverly, MA, USA).

### 4.2. Cell Lines and Culture Conditions

The human breast cancer cell lines MDA-MB-231, MCF-7, BT-474, T-47D, and SKBR3 cells were purchased from ATCC (Rockville, MD, USA). Breast cancer cell lines were maintained in RPMI-1640 media supplemented with 10% FBS, 100 U/mL penicillin G, and 0.1 mg/mL streptomycin. All cell lines were maintained in a humidified atmosphere of 5% CO_2_ at 37 °C. Araguspongines and xestospongin B were first dissolved in a volume of DMSO to provide final 10 mM stock solutions which were used to prepare various concentrations of treatment media. Final concentration of DMSO was maintained as the same in all treatment groups within a given experiment and never exceeded 0.1%.

### 4.3. Measurement of Viable Cell Number

Viable cell count was determined using the 3-(4,5-dimethylthiazol-2yl)-2,5-diphenyl tetrazolium bromide (MTT) colorimetric assay [19]. The optical density of each sample was measured at 570 nm on a microplate reader (BioTek, Winooski, VT, USA). The number of cells/well was calculated against a standard curve prepared by plating various concentrations of cells, as determined using a hemocytometer at the start of each experiment.

### 4.4. Cell Viability Assays

Cells were plated at a density of 1 × 10^4^ cells per well (6 wells/group) in 96-well culture plates and maintained in RPMI-1640 media supplemented with 10% FBS and allowed to adhere overnight. The next day, cells were divided into different treatment groups and given various treatments in RPMI-1640 medium containing 40 ng/mL HGF. Viable cell number after 48 h treatment was determined using the MTT assay.

### 4.5. Soft Agar Assay

The colony-forming capacity of BT-474 breast cancer cells was assessed using the CytoSelect 96-Well *in Vitro* Tumor Sensitivity Assay (Soft Agar Colony Formation) Kit (Cell Biolabs Inc., San Diego, CA, USA), according to manufacturer’s protocol. Briefly, 50 μL of Base Agar Matrix Layer was dispensed into each well of a 96-well tissue culture plate. Cells (5 × 10^3^) in 75 μL of Cell Suspension/Agar Matrix Layer were dispensed into each well. The cells were treated with 50 μL of culture medium containing DMSO or 10 μM of araguspongine C. After 8 days’ incubation, visible colonies were photographed under an inverted phase-contrast microscope.

### 4.6. Western Blot Analysis

Cells were treated according to the methods described previously [19]. At the end of the treatment period, cells were lysed in RIPA buffer (Qiagen Sciences Inc., Valencia, CA, USA) and protein concentration was determined by the BCA assay (Bio-Rad Laboratories, Hercules, CA, USA). Equivalent amounts of protein (30 μg) were electrophoresed on SDS–polyacrylamide gels. The gels were then electroblotted onto PVDF membranes. These PVDF membranes were then blocked with 2% BSA in 10 mM Tris-HCl containing 50 mM NaCl and 0.1% Tween 20, pH 7.4 (TBST) and then, incubated with specific primary antibodies overnight at 4 °C. At the end of the incubation period, membranes were washed 5 times with TBST and then incubated with respective horseradish peroxide-conjugated secondary antibody in 2% BSA in TBST for 1 h at room temperature followed by rinsing with TBST for 5 times. Blots were then visualized by chemiluminescence according to the manufacturer’s instructions (Pierce, Rockford, IL, USA). Images of protein bands from all treatment groups within a given experiment and scanning densitometric analysis were acquired using Kodak Gel Logic 1500 Imaging System (Carestream Health Inc., New Haven, CT, USA). All experiments were repeated at least three times.

### 4.7. Apoptosis Analysis with Annexin V Staining by Flow Cytometry

Induction of apoptosis was assessed by the binding of annexin V to phosphatidylserine, which is externalized to the outer leaflet of plasma membrane early during induction of apoptosis. Analysis of annexin V was determined using Annexin V-FITC Early Apoptosis Detection Kit (Cell Signaling Technology, Beverly, MA, USA). BT-474 cells were plated at a density of 5 × 10^6^ cells/100 mm culture plates, allowed to attach overnight. Afterwards, cells were incubated in the respective control or araguspongine C treated with defined serum-free medium containing 40 ng/mL of HGF for 48 h. At the end of the experiment, cells in each treatment group were isolated with trypsin and then washed twice with ice cold PBS. Cells were then resuspended in 96 μL of ice-cold 1× Annexin V Binding Buffer. Afterwards, 1 μL Annexin V-FITC Conjugate and 12.5 μL Propidium Iodide (PI) Solution were added to each 96 µL cell suspension. The cells were then incubated for 10 min on ice in the dark. The cell suspension was then diluted to a final volume of 250 μL per assay with ice-cold, 1× Annexin V Binding Buffer. Dot plots were generated using CellQuest software (BD Biosciences, San Jose, CA, USA), and they were divided into 4 quadrants (LL: lower left; LR: lower right; UL: upper left; UR: upper right). The LL quadrant shows cells negative for both annexin V and PI (living, non-apoptotic cells). The LR quadrant shows cells positive for annexin V, but negative for PI (living, early apoptotic). The UL quadrant shows cells positive for PI, but negative to annexin V (dead), whereas the UR quadrant shows cells positive for both annexin V and PI (late apoptotic). All experiments were repeated at least three times.

### 4.8. Cyto-ID Staining Assay

Cyto-ID is a proprietary reagent specifically labels autophagic vacuoles and co-localizes with light chain 3 (LC3). Cyto-ID Autophagy detection kit (Enzo Life Sciences, Farmingdale, NY, USA) was used according to the manufacturer’s protocol. The fluorescence was measured by a plate reader (BioTek, Winooski, VT, USA). Cyto-ID Green reagent staining showed the relative fluorescence intensity of cells was increased in a dose-dependent manner, indicating the occurrence of autophagy. Rapamycin was used as a positive control [39].

### 4.9. Z-LYTE c-Met Kinase Assay

Z-LYTE Kinase Assay-Tyr6 Peptide kit (Invitrogen, Carlsbad, CA, USA) was used to assess the ability of araguspongines A and C to inhibit c-Met phosphorylation. Briefly, 20 μL/well reactions were set up in 96-well plates containing kinase buffer, 200 μM ATP, 4 μM Z-LYTE Tyr6 Peptide substrate, 2500 ng/mL c-Met kinase and compound of interest as an inhibitor. After 1 h of incubation at room temperature, 10 μL development solution containing site-specific protease was added to each well. Incubation was continued for 1 h. The reaction was then stopped, and the fluorescent signal ratio of 445 nm (coumarin)/520 nm (fluorescein) was determined on a plate reader (BioTek, Winooski, VT, USA) which reflects the peptide substrate cleavage status and/or the kinase inhibitory activity in the reaction.

### 4.10. Molecular Modeling

The *in-silico* experiments were carried out using Schrödinger molecular modeling software package installed on an iMac 27-inch Z0PG workstation with a 3.5 GHz Quad-core Intel Core i7, Turbo Boost up to 3.9 GHz, processor and 16 GB RAM (Apple, Cupertino, CA, USA).

#### 4.10.1. Protein Structure Preparation

The X-ray crystal structure of the human c-Met kinase domain; residues 1048–1350, (PDB code: 3IN5, [41]) was retrieved from the Protein Data Bank [42]. The Protein Preparation Wizard of the Schrödinger suite was implemented to prepare the c-Met kinase domain [43]. The protein was reprocessed by assigning bond orders, adding hydrogens, creating disulfide bonds and optimizing H-bonding networks using PROPKA (Jensen Research Group, Copenhagen, Denmark). Finally, energy minimization with a root mean square deviation (RMSD) value of 0.30 Å was applied using an Optimized Potentials for Liquid Simulation (OPLS_2005, Schrödinger, New York, NY, USA) force field.

#### 4.10.2. Ligand Structure Preparation

The structure of each araguspongine was sketched in the Maestro 9.3 panel (Maestro, version 9.3, 2012, Schrödinger, New York, NY, USA). The Lig Prep 2.3 module (Lig Prep, version 2.3, 2012, Schrödinger, New York, NY, USA) of the Schrödinger suite was utilized to generate the 3D structure and to search for different conformers. The Optimized Potentials for Liquid Simulation (OPLS_2005, Schrödinger, New York, NY, USA) force field was applied to geometrically optimize the ligand and to compute partial atomic charges. Finally, at most, 32 poses per ligand were generated with different steric features for the subsequent docking studies.

### 4.11. Molecular Docking

The prepared X-ray crystal structure of c-Met kinase domain was employed to generate receptor energy grids using the default value of the protein atomic scale (1.0 Å) within the cubic box centered on the cocrystallized ligand. After receptor grid generation, the prepared ligands were docked using the Glide 5.8 module (Glide, version 5.8, 2012, Schrödinger, New York, NY, USA) in extra precision (XP) mode [44].

### 4.12. Statistics

The results are presented as means ± SEM of at least three independent experiments. Differences among various treatment groups were determined by the analysis of variance (ANOVA) followed by Dunnett’s test using PASW statistics^®^ version 18. A difference of *P* < 0.05 was considered statistically significant as compared to the vehicle-treated control group. The IC_50_ values (concentrations that induce 50% cell growth inhibition) were determined using non-linear regression curve fit analysis using GraphPad Prism software version 6.

## 5. Conclusions

This is the first study to comprehensively characterize and evaluate the anticancer properties of the oxaquinolizidine alkaloids in breast cancer models. Results showed that araguspongine C-induced suppression of BT-474 cancer cell growth is mediated by induction of autophagic cell death. Autophagic activity of araguspongine C was associated with downregulation of c-Met and HER2 RTKs and suppression of receptor activation (Figure 9). Inhibition of these receptors was further confirmed by cell-free kinase assays and *in-silico* docking analysis. Suppression of RTK activity was also linked to PI3K/Akt/mTOR pathway inhibition (Figure 9). In addition, araguspongine C caused a reduction in the expression levels of IP3 receptor. Collectively, these results support the potential of araguspongine C as an inhibitor for RTKs and provide evidence for its anticancer activity in mammary tumor cells.

## Abbreviations

ER: estrogen receptor
EGFR: epidermal growth factor receptor
HER-2: human epidermal growth factor receptor 2
HGF: hepatocyte growth factor
PDB: protein data bank
H & E: hematoxylin and eosin
ATCC: American Type Culture Collection
RPMI: Roswell Park Memorial Institute
DMSO: dimethyl sulfoxide
FBS: fetal bovine serum
RIPA: radioimmunoprecipitation assay
BCA: bicinchoninic acid assay
PVDF: polyvinylidene fluoride
BSA: bovine serum albumin
NIH: National Institutes of Health
HRP: horseradish peroxidase
MAPK: mitogen-activated protein kinase
PI3K: phosphatidylinositol 3-kinase
PLC-γ: phospholipase C-γ
STATs: transcription factors like the signal transducers and activators of transcription
